# A new, genetically divergent species of *Pseudobaikalia* Lindholm, 1909 (Caenogastropoda, Baicaliidae)

**DOI:** 10.3897/zookeys.593.8511

**Published:** 2016-05-26

**Authors:** Tatiana Sitnikova, Maria Kovalenkova, Tatiana Peretolchina, Dmitry Sherbakov

**Affiliations:** 1 Limnological Institute SBRAS, P.O. Box 664033, Irkutsk, Russia; 2 Irkutsk State University, P.O. Box 664011, Irkutsk, Russia

**Keywords:** Gastropoda, new species, Pseudobaikalia michelae, Lake Baikal, Siberia

## Abstract

A new gastropod species, *Pseudobaikalia michelae* Sitnikoiva & Kovalenkova, **sp. n.**, (family Baicaliidae) is described from Lake Baikal. This is the first new species from the Baicaliidae for forty years. The new species is distinguished from its sister taxa by means of comparative morphology as well as analyses of DNA sequences (mtDNA and an intron of alpha-subunit gene of ATP-synthase). It was found in the southern and central-eastern parts of the lake where it occurs sympatrically with three other baicaliid species. Characters of the female reproductive system (i.e., a long oviduct loop with 2–3 narrow tube-like evaginations) and the aperture (i.e., oval shape with a simple outer lip) place these snails in the genus *Pseudobaikalia* Lindholm, 1909. The new species is most similar in its shell morphology to the northern Baikal species *Pseudobaikalia jentteriana* (smooth elongated shape) but differs by a more oval aperture that is slightly angled to the columella. Combined mitochondrial and nuclear sequences in a Bayesian analysis showed that all specimens of *Pseudobaikalia michelae* **sp. n.** form a well-supported clade.

## Introduction

Lake Baikal is the most ancient freshwater lake on Earth. It is inhabited by very diverse endemic species flocks (Sherbakov 1999). The family Baicaliidae Fisher, 1885 (Radoman 1983, Sitnikova et al. 2004) or subfamily Baicaliinae of the family Amnicolidae (Bouchet et al. 2005, Kantor et al. 2010) includes eight genera and nearly 40 species (Dybowski 1875, Lindholm 1909, Kozhov 1936, Sitnikova et al. 2004). The last new species description for this taxon dates back to 1975 (Beckman and Starobogatov 1975), more than 40 years ago. At present, nucleotide sequences for mitochondrial and nuclear genes are known for 23 species of baicaliids (Zubakov et al. 1997, Peretolchina et al. 2008, Kovalenkova et al. 2015). Molecular analyses of baicaliids have revealed limited phylogenetic structure based on mtDNA sequences (Zubakov et al. 1997). In this study the phylogeny inferred from sequences of cytochrome c oxidase subunit I (CO1), mitochondrial small subunit rDNA (mtSSU) and intron of alpha-subunit gene of ATP-synthase confirms that specimens of *Pseudobaikalia michelae* sp. n. form a separate clade. Indeed, unique conchological and anatomical characters of these snails add support to their status as a new species.

## Material and methods

**Figure 1.**
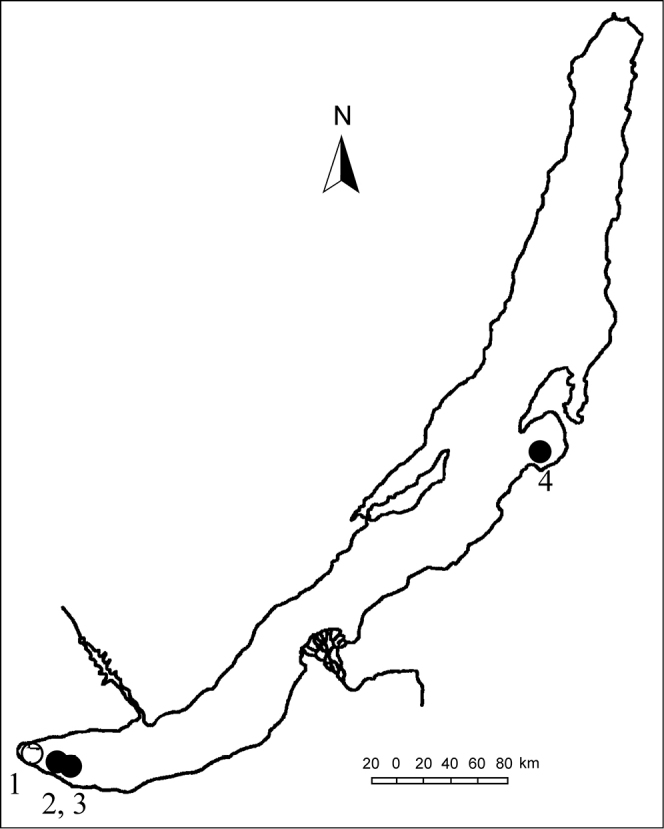
The location of *Pseudobaikalia michelae* sp. n. sampling sites in Lake Baikal. Site numbers correspond to **1** Kultuk Bay **2** near Utulik settlement **3** Murinskaya Bank **4** Barguzin Bay.

The type material of *Pseudobaikalia michelae* sp. n. was collected by dredging during sampling expedition to Lake Baikal on 11 October 2009. Additionally, two specimens of the new species were defined among syntypes of *Pseudobaikalia pulla pulla* (=Leucosia angarensis var. pulla W. Dybowski, 1875) collected by Benedict Dybowski, probably near Kultuk settlement, place of his political exile, and hosted in the freshwater gastropod collection of Zoological Institute RAS (Saint Petersburg). Some snails were found at three further sites of the Lake (Fig. 1) and stored in Limnological Institute SB RAS (Irkutsk).

**Figure 2.**
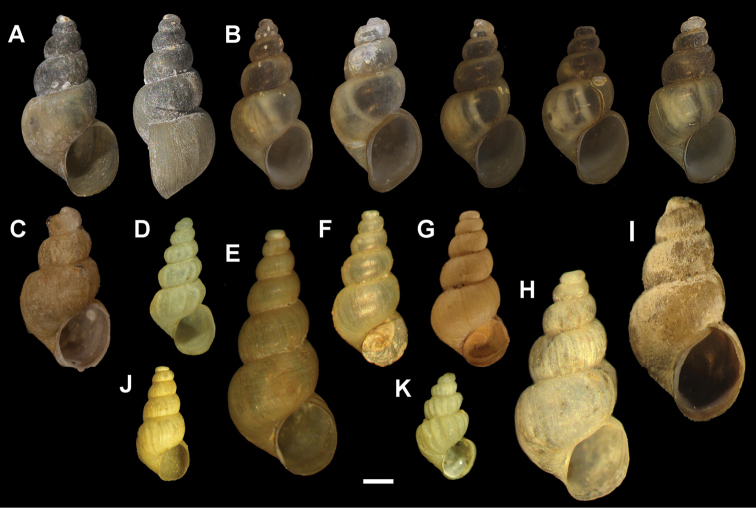
Shells of the type specimens of *Pseudobaikalia michelae* sp. n. and syntypes of other species of *Pseudobaikalia* genus from ZIN collection. **A** Holotype *Pseudobaikalia michelae* sp. n. **B** Paratypes *Pseudobaikalia michelae* sp. n. used for analyses **C** *Pseudobaikalia michelae* sp. n. (=Leucosia angarensis var. pulla, det. Dybowski, No. 1) **D** *Pseudobaikalia elegantula* (No. 2) **E** *Pseudobaikalia jentteriana* (No. 1) **F** *Pseudobaikalia pulla pulla* (No. 1) **G** *Pseudobaikalia pulla tenuicosta* (No.1) **H** *Pseudobaikalia zachwatkini* (No. 1) **I** *Parabaikalia elata* (=*Baikalia angarensis elata*, No.1) **J** *Pseudobaikalia cancellata* (No. 1) **K** *Pseudobaikalia contabulata* (No. 1). Scale bar 1 mm.

The representatives of other species of baicaliids used for molecular analyses were collected at different sites of the lake during expeditions in 2009–2014 by dredging or diving (for details see Table 1). The shells of the new species were compared to type specimens of baicaliids (Fig. 2D–K) housed in the collection of the Zoological Institute RAS.

**Table 1.** Collection localities and GenBank accession numbers for specimens included in the molecular analysis; n=number of specimens.

Taxon	Locality, depth in meters, substrate (collection date)	GenBank Accession No.		
		CO1	16S rDNA	Intron ATP α
*Parabaikalia elata* (n=2)	Peschanaya Bay, 8.6 m, sand (29.07.09)	KT885122	–	KF201704
*Pseudobaikalia contabulata* (n=2)	Kurkut Bay, 5 m, sand (27.08.11); Olkhon Gate, 15 m, sand (03.09.12)	KT808643, KT808642	KT885135, KT885136	KT885109, KT885134
*Pseudobaikalia jentteriana* (n=2)	Olkhon Gate, 37–38 m, sand, silt (13.10.09)	KT808645, KT885125	KT885137, KT885138	KT885104, KT885105
*Pseudobaikalia michelae* sp. n. (n=6)	Kultuk, 11–27 m, stones, coarse sand (11.10.09)	KT808639 – KT808641, KT885126 – KT885128	KT885139 – KT885144	KT885096 – KT885101
*Pseudobaikalia pulla pulla* (n=2)	Listvyanka, 10–14 m, sand, silt (06.02.14)	KT885129	KT885145	KT885107
*Pseudobaikalia pulla tenuicosta* (n=3)	Olkhon Gate, 37–38 m, sand, silt (13.10.09); Onokachanskaya, 10–15 m, sand (24.09.13)	KT808646, KT808648	KT885146, KT885147	KF201700, KT885108
*Pseudobaikalia zachwatkini* (n=2)	Listvyanka, 10–14 m, sand, silt (06.02.14)	KT885130	KT885148	KT885095

Anatomical study and molecular analysis were performed using snails initially fixed in 80% ethanol and then stored in 70% ethanol after one day. Seven snails were photographed and then shells of six individuals were destroyed for dissection. Micrographs of protoconchs and radulae were produced using a SEM while soft tissues were dissected under a light stereomicroscope. Morphological study and descriptive terminology are based on the review of morphological characters of hydrobioid gastropods (Radoman 1983, Hershler and Ponder 1998). Measurements of the shells, radular teeth and inner organs were performed using the Image-Pro-Plus program for Windows XP.

DNA was extracted from muscle tissue from the molluscan foot by using the modified method of Doyle (Doyle and Doyle 1990). We used universal pairs of primers for amplification and sequencing fragments of the mitochondrial genes CO1 (Folmer et al. 1994), and mtSSU (Katana et al. 2001), as well as segment of the nuclear gene of ATP synthetase alpha-subunit containing a single intron (ATPase α) (Jarman et al. 2002). Polymerase chain reaction (PCR) amplifications (35 cycles) were carried out using a BioRad T100 Thermal Cycler under the following conditions: pre-denaturation of DNA at 94 °C for 2 min, denaturation of DNA at 94 °C for 20 s, primer annealing 1 min at 48 °C for CO1, 50 °C for mtSSU rDNA and 54 °C for intron, and nucleotide chain elongation at 72 °C for 1 min (+ 5 min in the last cycle). The amplification products were sequenced at JSC Sintol (Moscow). The nucleotide non-coding sequences were aligned taking into account their putative secondary structures using Mafft v. 6 (Katoh et al. 2009).

Localities and GenBank accession numbers for sequenced species are given in Table 1.

Sites of mtSSU rDNA and ATPase α containing indels were excluded from the analysis. Concatenated 1529 bp long fragments (618 bp of mtDNA CO1, 445 bp of mtSSU rDNA, and 466 ATPase α) were used for phylogenetic inferences despite the lack of ATPase α data for the outgroup taxa. mtSSU rDNA data was also missing for *Parabaikalia elata*. *Benedictia baicalensis* (Benedictiidae: Caenogastropoda) was used as the closest available outgroup based on a previous phylogenetic analysis (Hausdorf et al. 2003), although the substitutions at the third codon position in CO1 may potentially impede the inferences. The nucleotide sequences of CO1 and mtSSU rDNA for *Benedictia baicalensis* were taken from GenBank (accession numbers AF445330 and AF445339).

JModelTest v. 2.0.2 (Guindon and Gascuel 2003, Darriba et al. 2012) was used to find the appropriate substitution model by means of the Bayesian Information Criterion. The phylogenetic tree was inferred by the Bayesian total evidence method as implemented in MrBayes 3.2.1 (Ronquist et al. 2012). Four replicate analyses of four simultaneous chains (1 cold) were run for 6,000,000 generations, sampling trees every 100 generations; the first 30% of trees were discarded. Trees sampled from the stationary phase of each replicate analysis were pooled to construct a single 50% majority rule consensus tree with Bayesian posterior probabilities. Uncorrected genetic distances (*p*-distances) were computed in the package APE (Paradis et al. 2004) using R (R Core Team 2015).

## Results

### Taxonomy Family BAICALIIDAE Fisher, 1885

#### Genus PSEUDOBAIKALIA

Taxon classificationAnimaliaLittorinimorphaBaicaliidae

Lindholm, 1909

Baikalia (Pseudobaikalia) Lindholm, 1909: 42. Type-species: Baikalia jentteriana 1909 (by original designation).
Baicalia (Pseudobaicalia) : Kozhov 1936: 85 (type species Baikalia contabulata Dybowski, 1875).
Pseudobaikalia : Sitnikova 1991: 285 (female reproductive system morphology); Sitnikova et al. 2004: 947 (type species Baicalia jentteriana, species composition); Kantor et al. 2010: 28 (type species Baikalia jentteriana, list of species).

##### Diagnosis.

Shell elongated, height up to 10 mm at 5–6 well rounded or shouldered whorls, smooth or with transverse fine ribs, oval aperture with simple evenly rounded outer lip, without umbilicus, protoconch discoidal, lateral radular teeth with square face, its width equal to length of outer wing; length of capsule gland equal to albumen gland, loop of oviduct long, reaching the proximal end of albumen gland, oviduct loop a cluster that includes from 2–7 ‘tube-like evaginations’, sometimes beyond the albumen gland.

##### Remarks.

The earlier diagnosis of genus (subgenus) *Pseudobaikalia* involved only conchiological traits (Lindholm 1909, Kozhov 1936) or morphology of reproductive system (Sitnikova 1991). Presently, the morphological details obtained from our study of *Pseudobaikalia michelae* sp. n. and early published data on radular teeth (Kozhov 1936) and protoconchs (Sitnikova et al. 2001) conform to the emended diagnosis of the genus *Pseudobaikalia*. Besides *Pseudobaikalia michelae* sp. n. the genus includes *Baikalia jentteriana*, Lindholm, 1909, *Baikalia contabulata* (Dybowski, 1875), *Baikalia pulla pulla* (Dybowski, 1875) (= *Baikalia subcilindrica* Lindholm, 1909), *Pseudobaikalia pulla tenuicosta* (Lindholm, 1909), *Pseudobaikalia elegantula* (Lindholm, 1909), *Pseudobaikalia cancellata* (Lindholm, 1909), and *Pseudobaikalia zachwatkini* (Kozhov, 1936). The shell photos of the types of these earlier described species are presented here for the first time (Fig. 2D–K).

#### Pseudobaikalia michelae

Taxon classificationAnimaliaLittorinimorphaBaicaliidae

Sitnikoiva & Kovalenkova sp. n.

http://zoobank.org/547D6538-64A8-4E90-8503-3048991BB626

**Figure 3.**
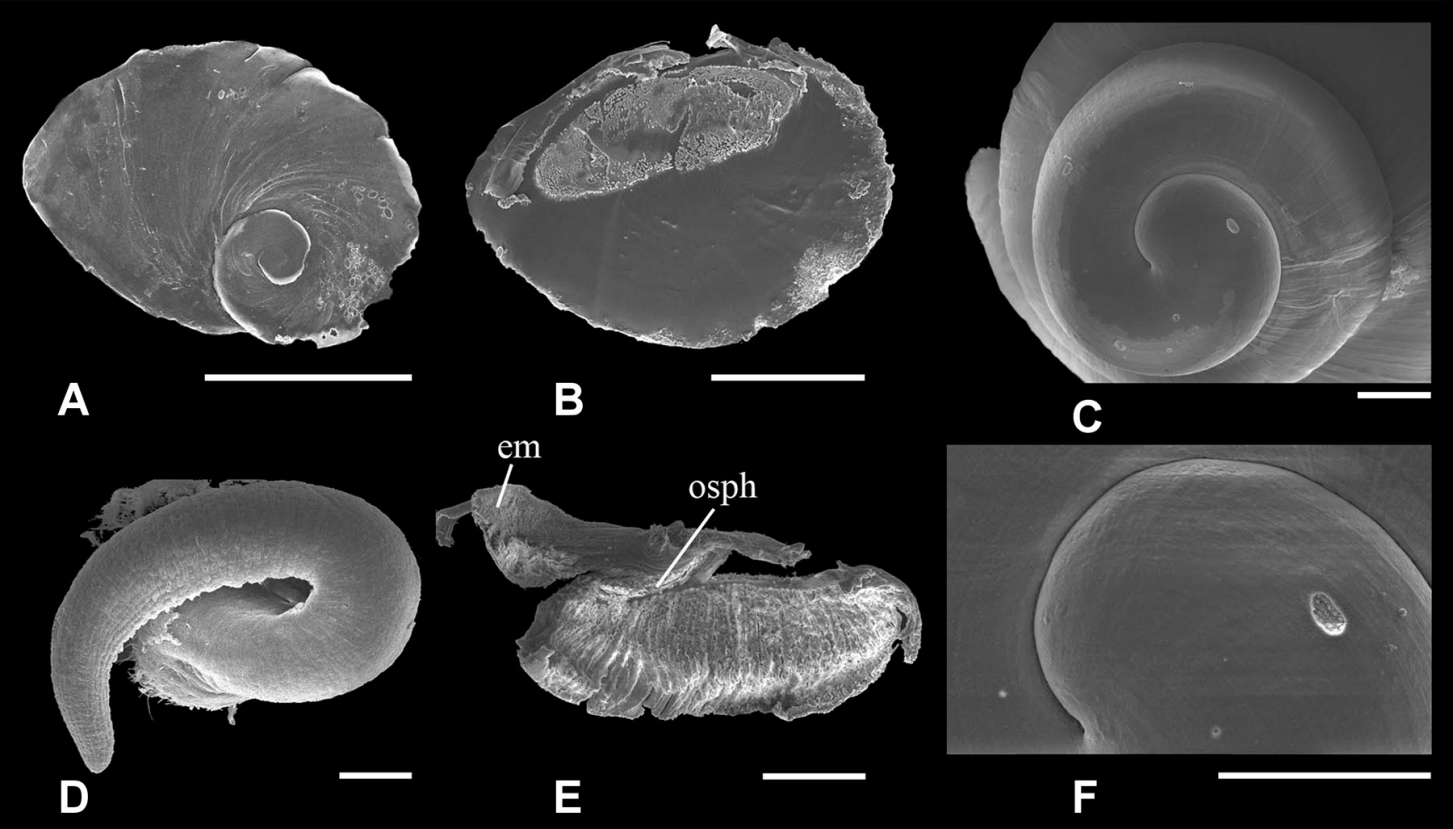
Scanning electron micrographs of *Pseudobaikalia michelae* sp. n. **A, B** Operculum, dorsal view to left and ventral view to right **C, E** Protoconch **D** Penis, dorsal view. Scale bars: 0.5 mm (**A, B**); 0.1 mm (**C, D, E**).

**Figure 4.**
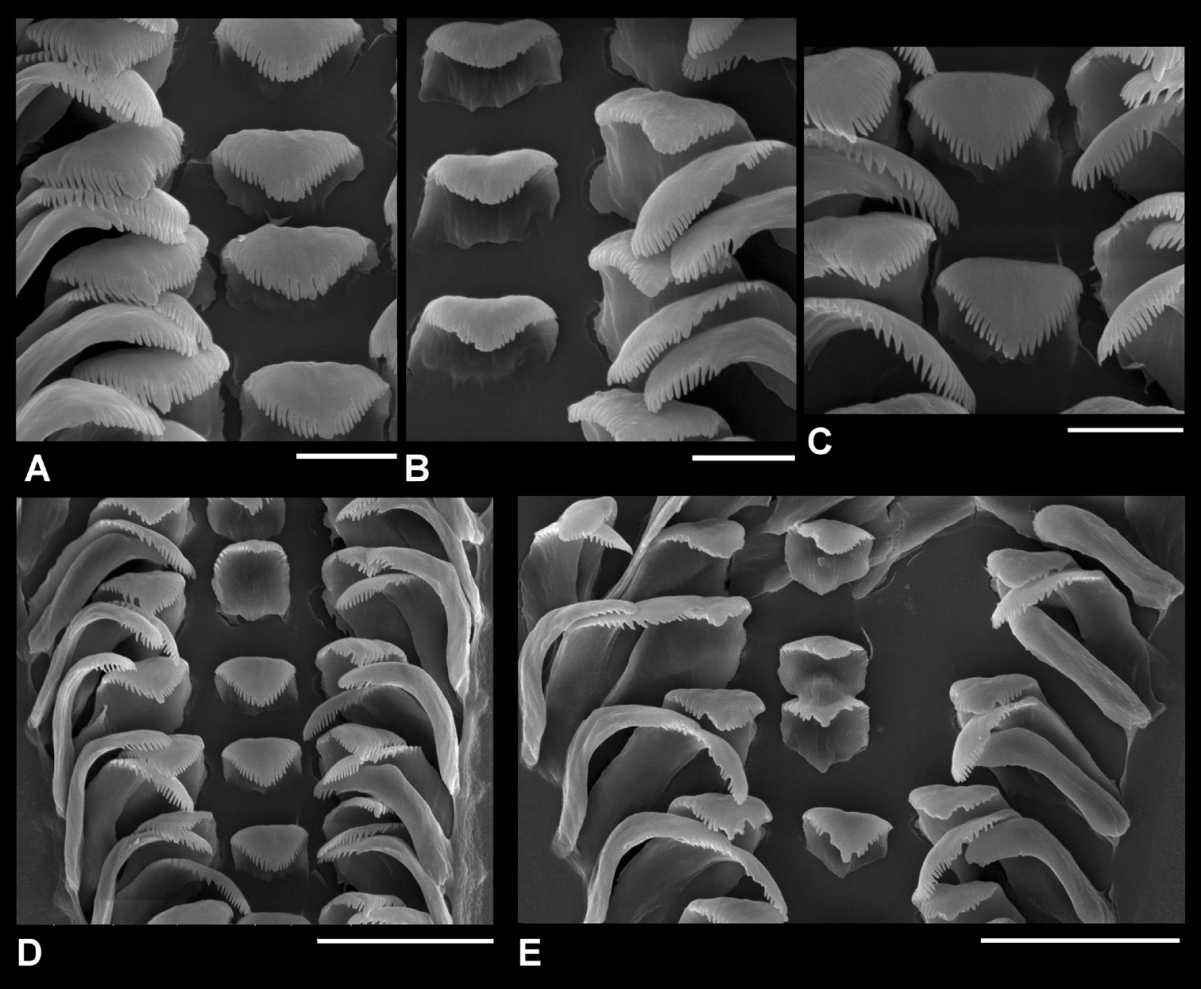
Radular teeth of *Pseudobaikalia michelae* sp. n. Scale bars: 10 μm (**A, B, C**); 30 μm (**D, E**).

**Figure 5.**
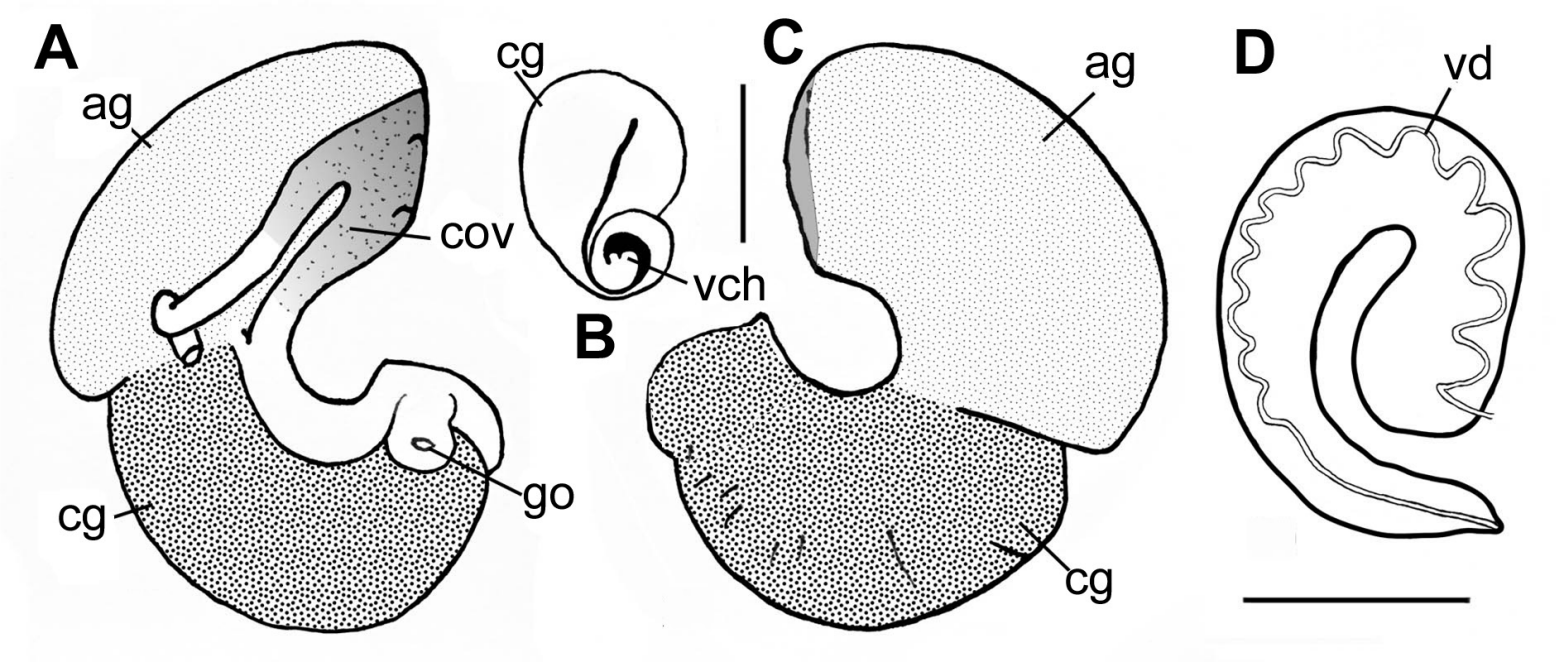
Genitalia of *Pseudobaikalia michelae* sp. n. **A, C** Ventral and dorsal view of female genitalia **B** Section of capsule gland and ventral channel **D** Penis. Abbreviations: ag—albumen gland; cg—capsule gland; cov—coil oviduct; go—genitalia opening; vch—ventral channel; vd—vas deference. Scale bars 0.5 mm.

Figs 2A, B, C , 3 , 4 , 5

##### Etymology.

The species name ‘*michelae*’ is in honour of Ellinor Michel (Natural History Museum, London) who has made a range of studies on gastropods inhabiting ancient lakes.

##### Type material.

Holotype (dry) and 2 paratypes (dry and in alcohol) from the type locality were deposited in freshwater gastropod collection of Zoological Institute RAS (Saint Petersburg, Russia) and are registered under Nos. 1/522–2015 (holotype) and 2/522–2015 (paratypes). Shells of six paratypes were destroyed for dissections (to study anatomy and nucleotide sequences) with images of them given here (Fig. 2B).

##### Additional material.

Two shells from a lot No. 1 of Zoological Institute collection with original label ‘Leucosia angarensis var. pulla, Baikal, collection W. Dybowski 1875’; 2 specimens: south-eastern part of Lake Baikal, near Utulik settlement (51°33'13.5"N, 104°05'17.3"E), at the depth of 17–31 m, silty sand; by drag, coll. by authors 07.21.2015 (site 2); 2 shells: the same part of the lake, Murinskaya Bank (51°30'11.3"N, 104°28'39.4"E), 34–39 m depth, stones, sand, silt; by drag, coll. by authors 07.21.2015 (site 3); 7 specimens: central part of the Lake, Barguzin Bay (53°18'34.8"N, 108°44'07.4"E), at a depth of 14 m, sand and stones; by drag, coll. by authors 07.17.2015 (site 4 in Fig. 1).

##### Type locality.

Kultuk Bay (southern part) of the Lake Baikal (southern Siberia), (51°42'59.9"N, 103°43'23.1"E) from 11 to 27 m depth, sand and stones, sponges (site 1, Fig. 1).

##### Description.

(Figs 2–5): Shell (Fig. 2A, B) grey-green or light brown, elongate-conic, smooth with 5–6 growth lines, small, up to 6.5 mm high and 3 mm wide; with 4.25–5.75 well rounded whorls, deep suture, without umbilicus; aperture elongate-oval, columellar lip slightly thickened, outer lip thin, simple or slightly rounded, basal lip rounded or slightly elongated. For shell dimensions please see Table 2. Protoconch (Fig. 3C, F) discoidal, about 1.25–1.45 whorls, diameter about 500 μ, surface almost smooth, near suture with slight spiral threads or slightly reticulate. Operculum (Fig. 3A, B) flat, thin, transparent, paucispiral with 5–6 growth whorls, last half whorl weakly frilled, nucleus subcentral, attachment scar elongated oval occupies about 1/3 of operculum width.

**Table 2.** Shell dimensions and whorl counts of type material of *Pseudobaikalia michelae* sp. n. Abbreviation: SH—height of shell; SW—width of shell; AW—width of aperture; AL—length of aperture; SpH—height of spire; LW—height of last whorl; n—number of whorls. Measurements are in mm.

Specimen	SH	SW	AW	AL	SpH	LW	n
Holotype	6.33	3.40	1.90	2.60	2.13	4.20	5.5
Paratypes	5.72	3.04	1.65	2.52	1.55	4.17	4.5
	5.98	2.84	1.62	2.16	2.12	3.86	5.75
	5.64	2.86	1.61	2.32	1.93	3.71	4.75
	5.08	2.82	1.62	2.25	1.45	3.63	4.25
	5.70	2.93	1.71	2.26	1.90	3.80	4.5
	5.06	2.64	1.49	2.03	1.70	3.36	5
	4.87	2.88	1.58	1.85	1.67	3.20	3.75
Mean ± SD (n = 7)	5.64 ± 0.46	2.93 ± 0.24	1.66 ± 0.13	2.31 ± 0.20	1.83 ± 0.27	3.82 ± 0.30	5.05 ± 0.5
Shell from ZIN collection	5.41	2.95	1.64	2.23	1.69	3.72	(3.5)

Radula (Fig. 4): 580–600 μm length with 46–48 teeth rows, 30–33 of them well-formed. Central teeth square or triangular, about 15.5 μm wide, cutting edge broadly concave; central cusp absent or square formed by the merger of 2–3 cusps; lateral cusps 15–16, thin and long; basal tongue slightly convex or straight. Lateral teeth face rectangular, outer margin with concave bend, central cusp similar to lateral cusps or slightly broader, or merger of 2–3 cusps, inner and outer lateral cusps about 9–10; outer wing rather broad, straight, about two times longer than cutting edge. Inner marginal teeth with approximately 24 cusps and outer marginal teeth with approximately 16 cusps.

Body pigmented black, mantle edge light grey, ctenidium nearly 2 mm in length with 62–64 leaflets, osphradium broadly ovate, slightly narrowed in proximal end, ~ 0.5 mm length, opposite anterior part of ctenidium, a little deeper than mantle fold.

##### Reproductive system.

Male with small bean prostate gland (Figs 5D, 3D), anterior and posterior vas deferens close together in middle part of prostate, penis light grey, muscular, elongated, with thin glandulous often non-visible fold, gradually tapering with short and small papilla. Female coiled oviduct with one spiral, loop grey pigmented with two ‘tube-like evaginations’ (Fig. 4A–C).

##### Ecology.

*Pseudobaikalia michelae* sp. n. was found on heterogeneous (stones, sand, and silt) or soft sediments at depth zone ranging from 11 to 39 m in southern, east-southern and central-eastern parts of Lake Baikal. At the type locality *Pseudobaikalia michelae* sp. n. was sympatric with three other species of baicaliids: *Godlewskia pulchella* (Dybowski, 1875), *Korotnewia semenkewitschi* Lindholm, 1909, and *Teratobaikalia duthiersii* Dybowski, 1875. Eleven baicaliids including *Pseudobaikalia michelae* sp. n. and three species of the genus *Pseudobaikalia*: *Pseudobaikalia contabulata*, *Pseudobaikalia pulla*, and *Pseudobaikalia zachwatkini* were collected together at sites 2 and 4 (Fig. 1); ten baicaliids were registered at site 3 (Fig. 1), including the species, *Pseudobaikalia contabulata* and *Pseudobaikalia zachwatkini*.

##### Remarks.

The genus *Pseudobaikalia* includes seven mainly shallow water species (Fig. 2D–K); some of them were found down to 100 or 200 m depth. Two species *Pseudobaikalia jentteriana* and *Pseudobaikalia cancellata* were found only in the northern part of the lake, *Pseudobaikalia elegantula* inhabits the northern and central parts of the lake, and three other species *Pseudobaikalia zachwatkini*, *Pseudobaikalia pulla*, and *Pseudobaikalia contabulata* are found in a range of locations throughout of Baikal, and are sympatric to *Pseudobaikalia michelae* sp. n.

**Figure 6.**
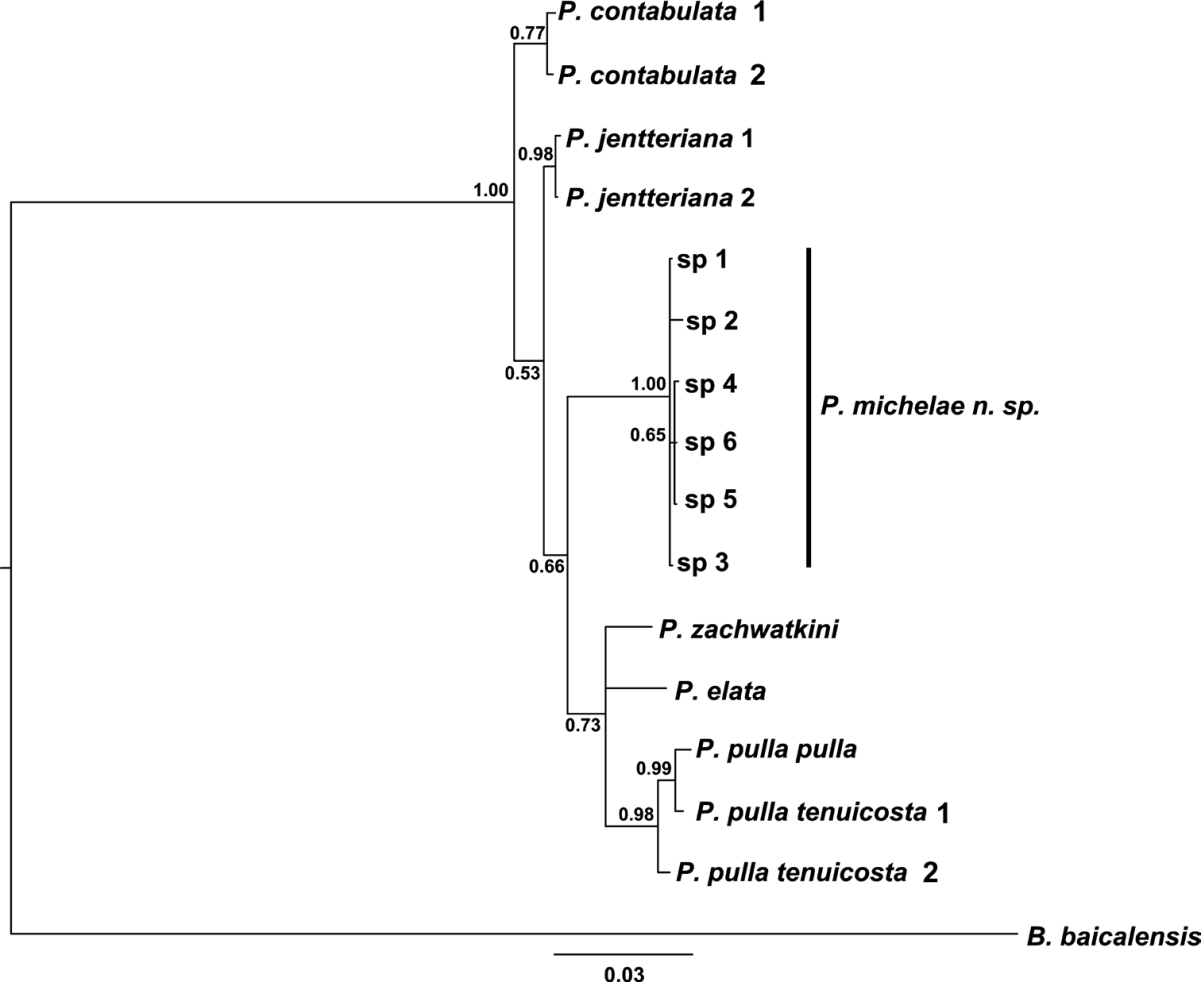
Phylogram derived from Bayesian analysis of combined CO1, 16S DNA and ATPase α gene intron sequences. Values at nodes represent posterior probabilities. The scale bar represents the branch length as a measure of substitution per site.

The shells of *Pseudobaikalia jentteriana* (Fig. 2E) and *Pseudobaikalia pulla pulla* (Fig. 2F) are smooth as in the new species, while the other *Pseudobaikalia* have transverse ribs or lirae. The shell of *Pseudobaikalia pulla tenuicosta* (from the northern part of Lake Baikal) has slightly raised ribs. Both subspecies of *Pseudobaikalia pulla* have an operculum equal to aperture size (Fig. 2F–G), but the operculum of other species including *Pseudobaikalia michelae* sp. n. is smaller than aperture. The new species is most similar to *Pseudobaikalia jentteriana* in its size and smooth shell but differs in colour (*Pseudobaikalia jentteriana* has light brown shell and body) and in the morphology of the female gonoduct (in *Pseudobaikalia jentteriana* the oviduct loop is wider and includes 3–4 ‘tube-like evaginations’); the penis of *Pseudobaikalia jentteriana* has not been investigated yet (Sitnikova 1991). The new species is similar to young *Parabaikalia elata* (Dybowski, 1975) in the shape of shell and aperture (Fig. 2I), but differs in adult shell size and the length of oviduct loop. *Parabaikalia elata* is partially sympatric to a new species, they co-occur at sites 3 and 4 (Fig. 1), and thus it was included into molecular analyses (Fig. 6, Table 3).

**Table 3.** Mean (uncorrected) CO1 (lower triangle) and intron ATP α (upper triangle) distances between *Pseudobaikalia michelae* sp. n. and other investigated species.

Taxon	*Parabaikalia elata*	*Pseudobaikalia contabulata*	*Pseudobaikalia pulla pulla*	*Pseudobaikalia pulla tenuicosta*	*Pseudobaikalia jentteriana*	*Pseudobaikalia zachwatkini*	*Pseudobaikalia michelae* sp. n.
*Parabaikalia elata*		0.015	0.022	0.023	0.014	0.015	0.013
*Pseudobaikalia contabulata*	0.057		0.029	0.030	0.021	0.022	0.020
*Pseudobaikalia pulla pulla*	0.036	0.060		0.010	0.010	0.011	0.013
*Pseudobaikalia pulla tenuicosta*	0.036	0.055	0.006		0.011	0.012	0.014
*Pseudobaikalia jentteriana*	0.057	0.009	0.059	0.052		0.004	0.007
*Pseudobaikalia zachwatkini*	0.039	0.050	0.040	0.038	0.047		0.007
*Pseudobaikalia michelae* sp. n.	0.078	0.048	0.070	0.067	0.048	0.074	

##### Molecular phylogeny.

Six specimens of *Pseudobaikalia michelae* sp. n. and thirteen specimens of sister species were analyzed using nucleotide sequences from the mitochondrial genes CO1, mtSSU rDNA and nuclear ATPase α intron segment.

JModelTest selected GTR + I + gamma for each CO1 codon positions separately HKY + I for mtSSU rDNA and HKY nucleotide substitution model for intron dataset as the best fit using the Bayesian Information Criterion.

Sequences of *Pseudobaikalia michelae* sp. n. cluster in the well-supported clade (posterior probability = 1.00) (Fig. 6). The average mutation distance from the new species to the other *Pseudobaikalia* species was found to be appropriate for species-level distinction: 6.4 ± 1.3%, 2.1 ± 0.2% and 2.1 ± 0.6% for the mitochondrial CO1 and mtSSU rDNA and the nuclear marker, respectively. Interspecies genetic distances are known for other taxa within this family, which can be non-monophyletic.

The lowest mitochondrial genetic distance between *Pseudobaikalia contabulata* and *Pseudobaikalia jentteriana* are comparable to the distances between representatives of subspecies of *Pseudobaikalia pulla*. They both are about 2% for CO1 and 0.03% for mtSSU rDNA. As for the CO1 data, *Pseudobaikalia michelae* sp. n. appears to be the sister group to *Pseudobaikalia contabulata* and *Pseudobaikalia jentteriana* with 4.8% of base substitutions. The minimum genetic distances in case of the intron of *Pseudobaikalia michelae* sp. n. is 0.7% to the two species: *Pseudobaikalia jentteriana* and *Pseudobaikalia zachwatkini* (Table 3). Moreover, there is an important higher-level character difference in the new species sequence profile as all intron sequences of *Pseudobaikalia michelae* sp. n. had a relatively large (57 BP) deletion.

## Discussion

The new species is well-differentiated from its sister species in the multigene phylogeny produced here. This is very unusual for Baicaliidae where species with a highly distinctive morphology may be distinguished from each other by very small genetic distances between them. On the other hand, the range of intra-specific polymorphism at least in case of *Baicalia carinata* exceeds the inter-specific distances between several species within this group of gastropods (Zubakov et al. 1997, Peretolchina et al. 2008).

Within *Pseudobaikalia* the interspecific mitochondrial distances are very small (about 1%) in case of *Pseudobaikalia jentteriana* and *Pseudobaikalia contabulata*. However, these two species are more distant from each other if estimated from the nuclear marker. Most likely it may be explained by secondary and possibly asymmetric hybridization events in the process of their speciation. Similar mechanisms might be responsible for the limited phylogenetic structure based on sequence data for Baicaliidae family as a whole. Thus the genetic separation of *Pseudobaikalia michelae* sp. n. from other *Pseudobaikalia* species appears to be an exception.

## Supplementary Material

XML Treatment for PSEUDOBAIKALIA

XML Treatment for Pseudobaikalia michelae
